# Supplementary material for: Environmental stiffness regulates neuronal maturation via Piezo1-mediated transthyretin activity
Source: Nat Commun. 2025 Nov 7;16:9842. doi: 10.1038/s41467-025-64810-3 (PMC12594958; doi:10.1038/s41467-025-64810-3)
Supplement: Supplementary file 1 — Supplementary Information [file 41467_2025_64810_MOESM1_ESM.pdf]

Supplementary Information

Environmental stiffness regulates neuronal maturation via Piezo1-mediated transthyretin activity

Eva Kreysing[1,2,3,4,*], Hélène O. B. Gautier[1], Sudipta Mukherjee[1,2,3],Katrin A. Mooslehner[1,2,3], Leila Muresan[1,5], Daniel Haarhoff[6], Xiaohui Zhao[7], Alexander K. Winkel[1], Tina Borić[2,3], Sebastián Vásquez-Sepúlveda[2,3], Niklas Gampl[2,3], Andrea Dimitracopoulos[1], Eva K. Pillai[1], Robert Humphrey[1,8], Ragnhildur Thóra Káradóttir[8,*], and Kristian Franze[1,2,3,*]

[1] Department of Physiology, Development and Neuroscience, University of Cambridge, Downing Street, Cambridge CB2 3DY, UK

[2] Medical Institute of Biophysics, Friedrich-Alexander-Universität Erlangen-Nürnberg, Kussmaulallee 2, 91054 Erlangen, Germany

[3] Max-Planck-Zentrum für Physik und Medizin, Kussmaulallee 2, 91054 Erlangen, Germany

[4] Warwick Medical School, University of Warwick, Coventry CV4 7AL, UK

[5] School of Computing and Information Science, Faculty of Science and Engineering, Anglia Ruskin University, CB1 1PT Cambridge, UK

[6] Makespace Cambridge Ltd, 16 Mill Lane, Cambridge, CB2 1RX, UK

[7] Department of Medicine, Cardiovascular Division, University of Cambridge, Heart and Lung Research Institute, CB2 0BB, Cambridge, UK

[8] Cambridge Stem Cell Institute, Jeffrey Cheah Biomedical Centre, University of Cambridge Biomedical Campus, Puddicombe Way, Cambridge CB2 0AW, UK

* Correspondence: Eva Kreysing (emk42@cam.ac.uk), Ragnhildur Thóra Káradóttir (rk385@cam.ac.uk), Kristian Franze (kf284@cam.ac.uk)

Supplementary Methods

Buffers and Solutions

Neuron culture media for Patch clamp and synapse staining experiments
150 ml Neurobasal
1.5 ml Glutamax
1.5 ml Penicillin-Streptomycin
3mL B-27 supplement
1.5 mL of N2
Supplemented with 2µM AraC for the first media change and 1µM AraC afterwards
filtered through top bottle filter with 220 nm pore size

Papain solution
2 ml HBSS
20 µl Penicillin-Streptomycin
40 units Papain
20 µl DNase I Type IV
10 µl L-Cysteine
filtered through filter with 220 nm pore size

Ovomuccoid
50 ml of HBSS
0.5 ml Penicillin-Streptomycin
25 mg Bovine Serum Albumin
0.5 ml DNase I Type IV
50 mg Trypsin inhibitor
dissolved for 1h at 37°C

Borate buffer
1.24g Boric Acid
1.9g Borox
400ml water
pH adjusted to 8.5
water added to reach total volume of 500ml
filtered through filter with 220 nm pore size

NB complete
150 ml Neurobasal
1.5 ml GlutaMAX
3 ml B-27
1.5 ml Penicillin-Streptomycin-Amphotericin B Mixture (PSF)
filtered with top bottle filter with 100 nm pore size

HBSS+
150 ml HBSS w/o Calcium, w/o Magnesium
1.5 ml PSF
filtered with top bottle filter with 100 nm pore size

Hibernate E+
Mix 100 ml Hibernate E
1 ml PSF
filtered with top bottle filter with 100 nm pore size

Magic RIPA Buffer
150 mM NaCl
1% Triton
0.5% Sodium deoxycholate
0.1% Sodium dodecyl sulfate (SDS)
50 mM Tris(Trizma base)
pH 8

TBS
200 mM Tris
1.37 M NaCl
pH to 7.6

TBST
200 mM Tris
1.37 M NaCl
0.5% Tween-20
pH to 7.6

Running buffer
NuPAGE™ MOPS SDS Running Buffer (20X) diluted in double distilled water = ddH$_2$0

Complete Transfer Buffer (1L)
50 ml NuPAGE Transfer Buffer (20x)
100 ml methanol
850 ml ddH$_2$O
1 ml NuPAGE antioxidant

10x Marc's Modified Ringer Solution (10x MMR)
584.4 g NaCl
14.9 g KCl
20.33 g MgCl$_2$6H$_2$O
29.4 g CaCl$_2$2H$_2$O
20 ml EDTA disodium salt 0.5M
119.4 g Hepes
fill up to 10L with ddH$_2$O
approximately 20 ml NaOH (used to pH to 7.8)
Buffer was aliquoted and autoclaved

AFM *Xenopus* Media
6.5 ml 10 x MMR
500 µl PSF
0.02 g MS222
fill up to 50 ml with ddH$_2$O
pH to 7.5

Exposed Brain Media (EBM)
5 ml 10x MMR
20 mg MS222
500 µl PSF
fill up to 50 ml
pH to 7.4

Anaesthetic *Xenopus* Media
80 mg MS222
198 ml 1x MMR
2 ml PSF
pH to 7.6

***Xenopus* Recovery Media**
1:1 Anaesthetic *Xenopus* Media, 0.1x MMR
Xenopus Rearing Media
0.1x MMR

PBT
PBS with 0.2% BSA and 0.1% Triton X

Supplementary Figures

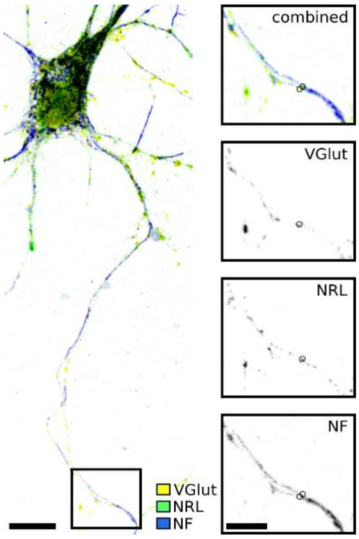

Supplementary Figure 1 | Detection of synapses The main panel shows a complete field of view (FOV) of neurons grown on a stiff substrate. The insets show the same area in the three channels relevant for this synapse: the presynaptic vesicular glutamate transporter (VGlut), postsynaptic neuroligin (NRL), and neurofilament (NF). Synaptic markers were identified to be co-localised if their intensity maxima (indicated by circles) were located within two pixels distance and colocalize with the NF signal. Scale bar in the main panel is 10 µm, in the insets 5 µm.

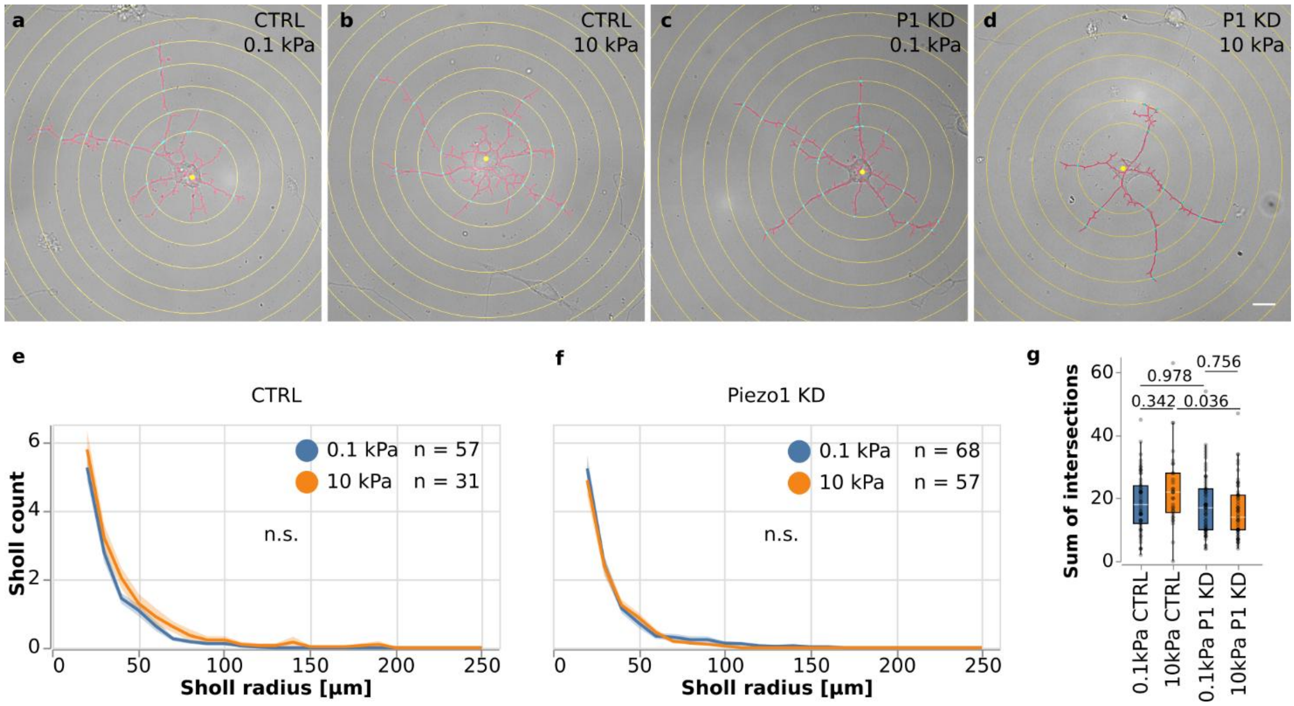

Supplementary Figure 2 | Quantification of neuronal arbor complexity. (a–d) Representative examples of skeletonised neurons. The number of intersections of neurites with concentric rings spaced by 10 µm (light blue) was quantified for control and Piezo1 KD cells, each cultured on both soft and stiff substrates ("Sholl analysis"). (e, f) Sholl profiles for (e) control and (f) Piezo1 knockdown neurons. Solid lines represent mean values, and shaded areas indicate the standard error of the mean. (g) For each cell, all neurite–ring intersections were quantified and compared across conditions using a two-way ANOVA with two-sided Sidak's post hoc test. Branching behaviour was unaffected by substrate stiffness in both CTRL and KD cells. P-values provided in the figure. Boxplots show the median (central line), the interquartile range (box); and whiskers represent 1.5 times the interquartile range. Scale bar: 10 µm.

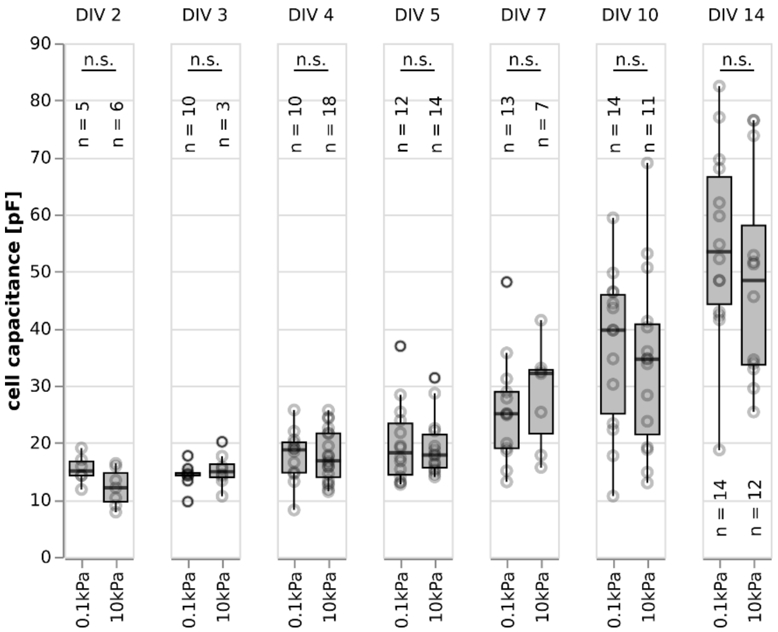

Supplementary Figure 3 | Cell capacitance as a function of time in culture and substrate stiffness. Between DIV 2-DIV 14, the capacitance increased over time on both substrates. It did not depend on substrate stiffness (two-tailed t-tests). Boxplots show the median (central line), the interquartile range (box), and whiskers represent 1.5 times the interquartile range.

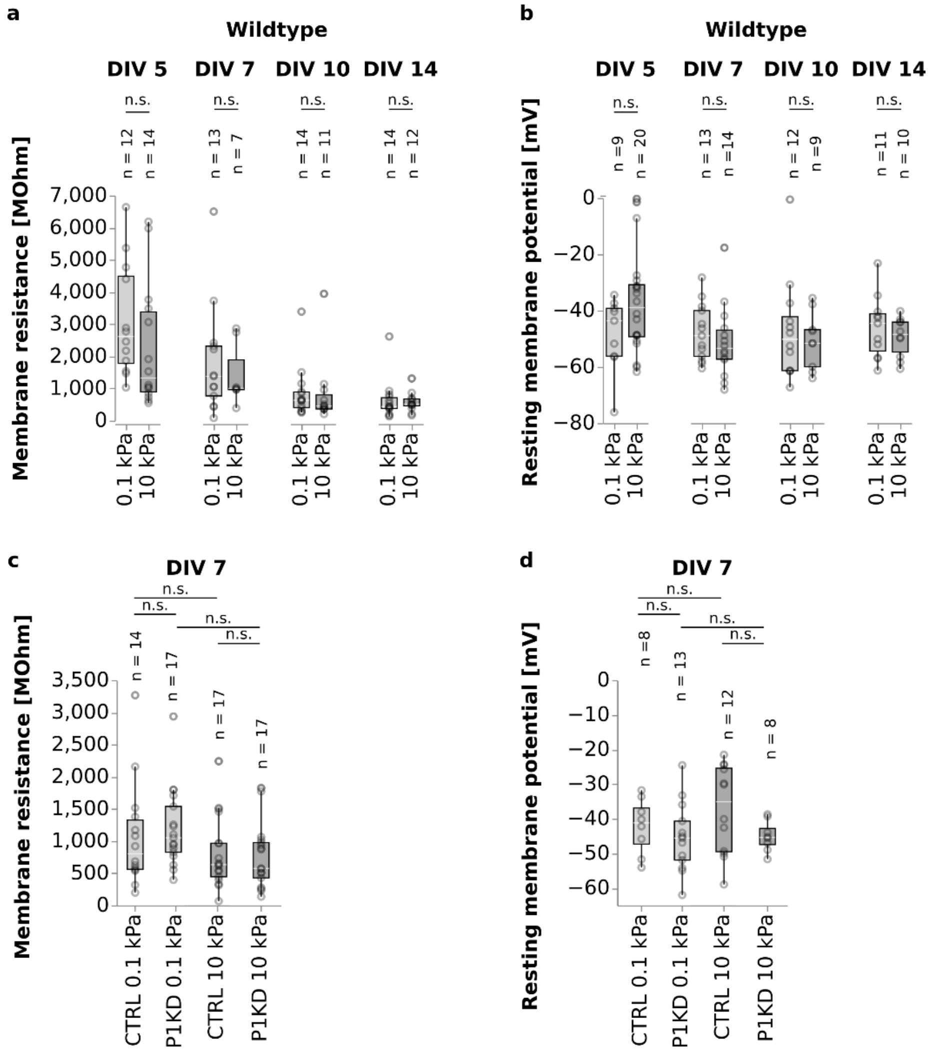

Supplementary Figure 4 | Membrane resistance and resting membrane potentials of wildtype (WT), Piezo1 knockdown (P1 KD) and control (CTRL) neurons on soft and stiff substrates. (a) Membrane resistance of WT neurons gradually decreased over the course of maturation, and was independent of substrate stiffness (two-tailed t-tests). (b) The resting membrane potential of neurons between DIV5 and DIV 14 was independent of substrate stiffness (two-tailed t-test). (c) Membrane resistance at DIV7 was independent of substrate stiffness and Piezo1 expression (two-way ANOVA with two-sided Sidak's post hoc test). (d) Resting membrane potential at DIV7 was independent of substrate stiffness and Piezo1 expression (two-way ANOVA with two-sided Sidak's post hoc test). Boxplots show the median (central line), the interquartile range (box), and whiskers represent 1.5 times the interquartile range.

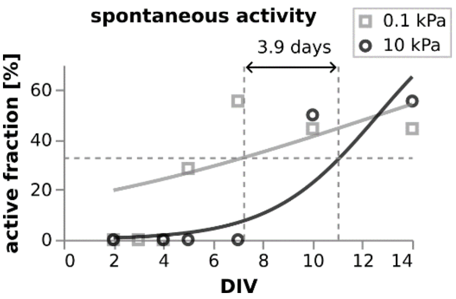

Supplementary Figure 5| Delay in the onset of spontaneous and evoked action potentials on stiff substrates. Logistic regression was used to estimate the delay in activity onset between substrates. The comparison was based on the time point at which each condition reached 50% of the final activity level observed in the soft substrate condition. Numbers of replicates are provided in Figure 2i.

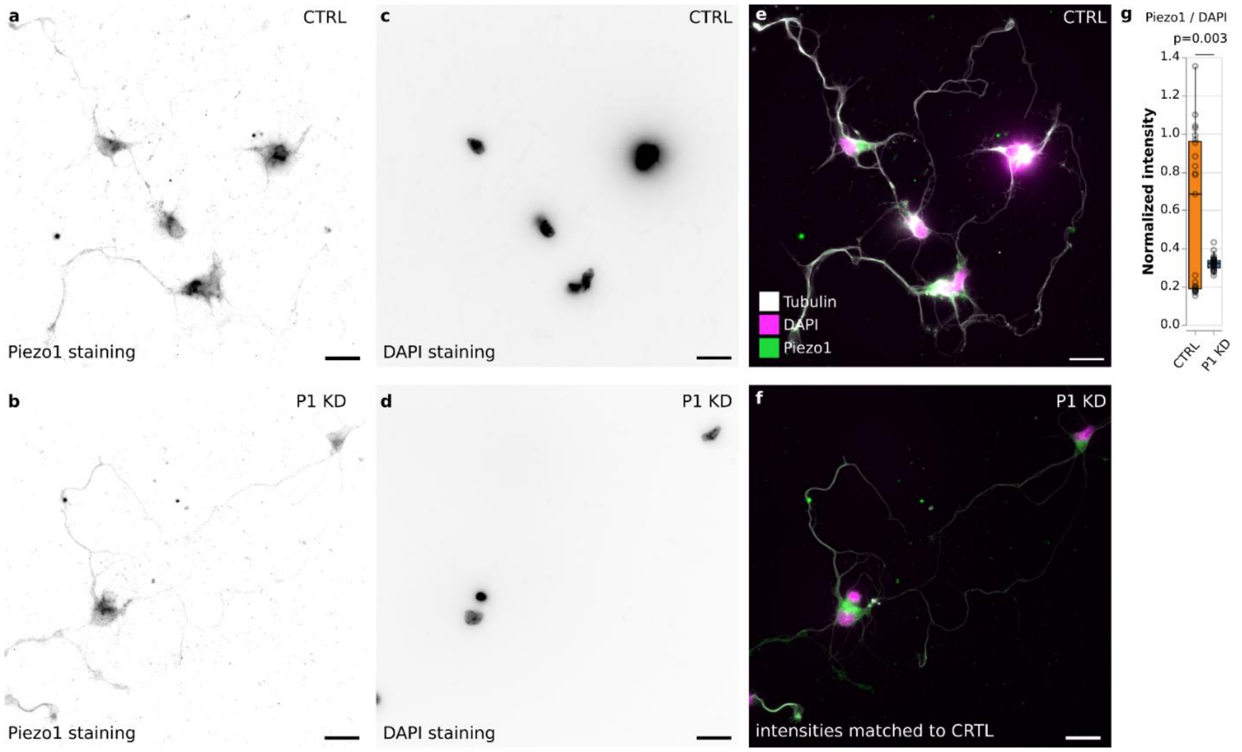

Supplementary Figure 6 | Characterization of Piezo1 KD in primary neurons. Stainings for individual proteins were intensity-matched between the two conditions. (a, c, e) CTRL neurons and (b, d, f) P1 KD neurons were stained for (a, b) Piezo1 and (c, d) nuclei (DAPI). (e, f) Piezo1, DAPI, and tubulin signals overlaid. (g) The intensities for each field of view (FOV) for the Piezo1 and the DAPI channels were calculated and normalised (see methods). Piezo1 expression was significantly reduced in the KD condition (two-tailed t-tests). For either condition, 23 FOVs were quantified. Boxplots show the median (central line), the interquartile range (box), and whiskers represent 1.5 times the interquartile range. Scale bars: 20 µm.

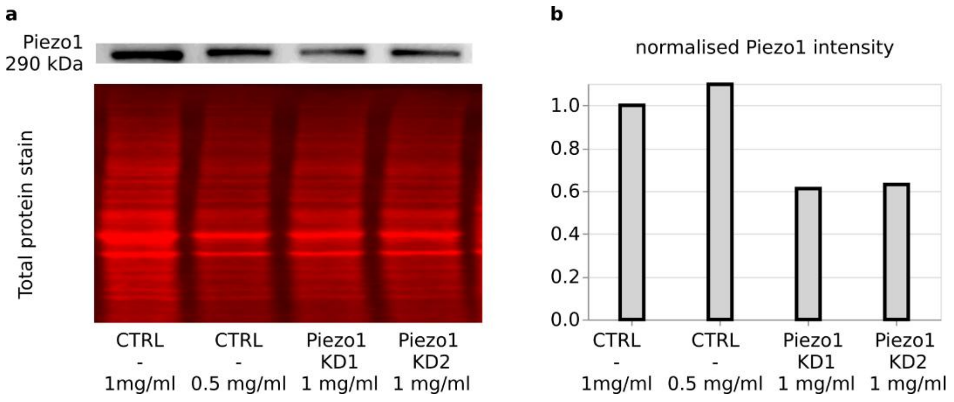

Supplementary Figure 7 | Western blots of CTRL and Piezo1 KD neurons. (a) Representative Western blots (WB) for the CTRL, Piezo1 KD1, and Piezo1 KD2 neurons. The protein lysates have been blotted at a protein concentration of 1mg/ml. For the CTRL cells, an additional lane has been run at 0.5 mg/ml protein concentration to check for linear behaviour of the WB quantification. The top row shows the Piezo1 stain, the bottom the total protein stain (TPS) used for normalisation. (b) Quantification of WBs. The normalised signals for CTRL cells are similar between 1 mg/ml and 0.5 mg/ml protein concentration, confirming the linearity of the signal. The CRISPR Cas9 Piezo1 KD conditions showed a signal reduction by about 40%.

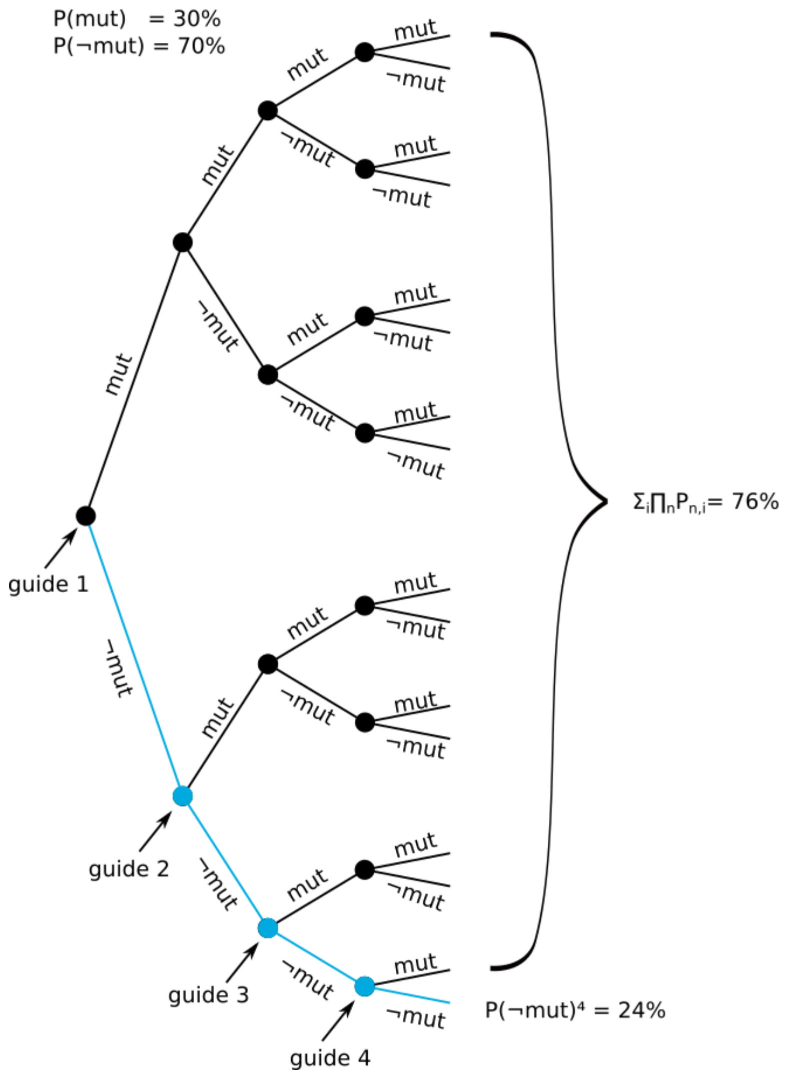

Supplementary Figure 8 | Probability tree for *Piezo1* guides introducing mutations in the *Piezo1*-gene. The probability for alterations in the *Piezo1* gene if 4 CRISPR/Cas9 guides are used simultaneously was calculated. It was assumed that each CRISPR guide has a 30% chance of successfully editing a gene and that the guides do not interfere with each other. For each individual gene, there is a 70% chance that a single guide will fail to edit the gene. So the probability that none of the four guides will edit the gene is 0.7^4 = 0.24 = 24%. This means that there is a 24% chance that the gene will remain completely unaltered by any of the guides. Vice versa, 76% of the time at least one of the guides will successfully edit the gene, resulting in mutations at one or more sites.

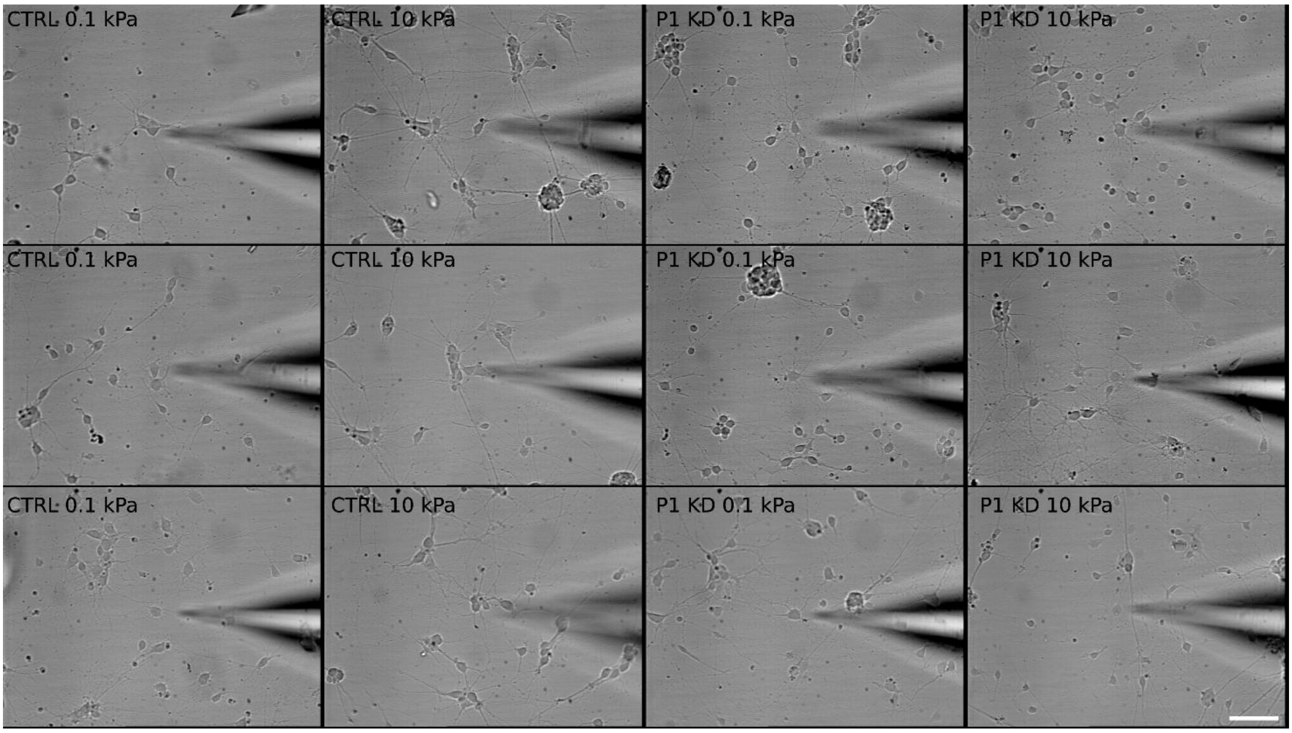

Supplementary Figure 9 | Representative brightfield images of control and Piezo1 knockdown (P1 KD) neurons at DIV7 on soft and stiff substrates. In each image, the patch pipette is visible on the right side, pointing to the neuron being recorded. Scale bar: 25 µm.

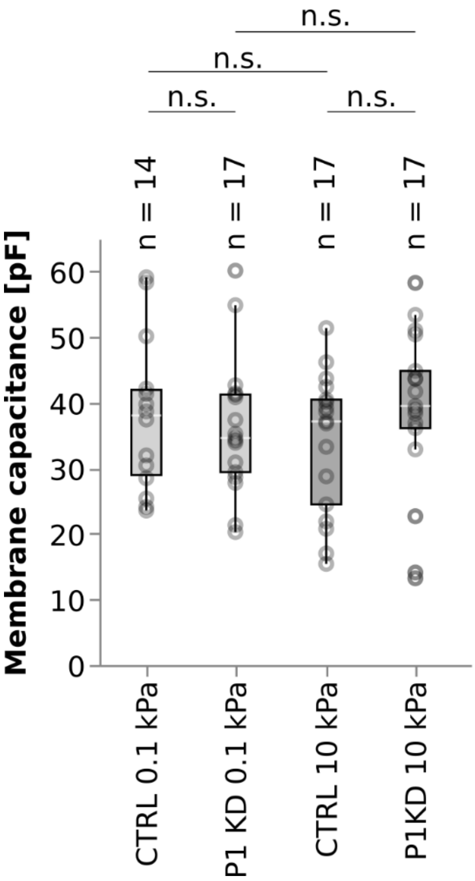

Supplementary Figure 10 | Membrane capacitance of control and Piezo1 KD cells. The capacitance of neurons at DIV 7 was independent of both substrate stiffness and Piezo1 expression (two-way ANOVA with two-sided Sidak's post hoc test). Boxplots show the median (central line), the interquartile range (box), and whiskers represent 1.5 times the interquartile range.

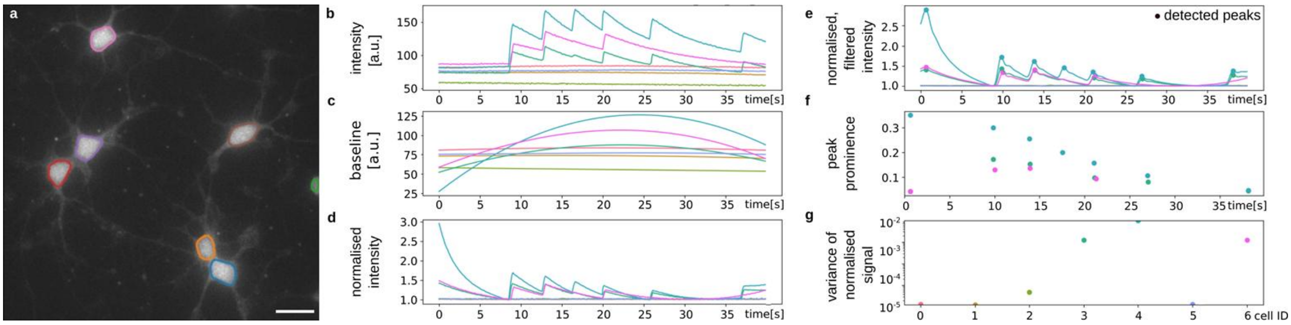

Supplementary Figure 11 | Step-by-step description of the peak detection algorithm. (a) FOV in calcium imaging series with outlined somata. (b) Raw intensity traces for each cell. (c) 3rd degree polynomial fit of the baseline for each trace. (d) The signals were normalised by dividing the raw signal by the baseline. (e) Signals after processing with Butterworth filter and detected peaks. (f) Peak prominence of each detected peak. (g) Peak height of each detected peak. Scale bar is 25 µm.

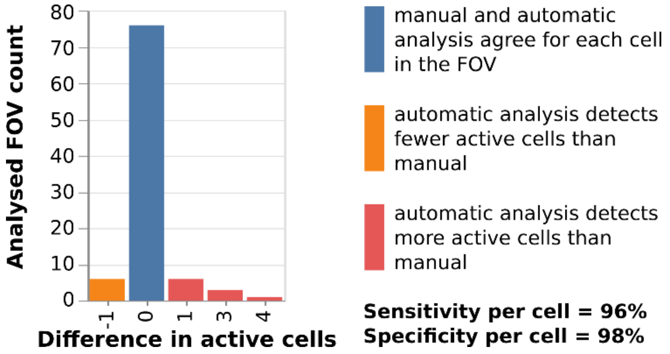

Supplementary Figure 12 | Quality check of the automated calcium analysis pipeline.

1281 cells from 92 FOVs were analysed manually and by the algorithm described in the Methods. We found a very good agreement between the two analysis approaches, indicating a high sensitivity and specificity of the automated detection of calcium peaks.

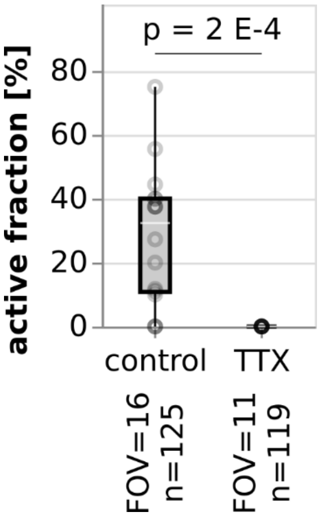

Supplementary Figure 13 | Perturbation of action potentials. The application of the neurotoxin Tetrodotoxin (TTX), which binds to voltage-gated sodium channels in neurons and thus inhibits action potentials, depleted the calcium transients observed in CTRL neurons cultured at DIV 7 **(**Mann-Whitney U test), indicating that the observed calcium transients were caused by the electrical activity of neurons. Boxplots show the median (central line), the interquartile range (box), and whiskers represent 1.5 times the interquartile range.

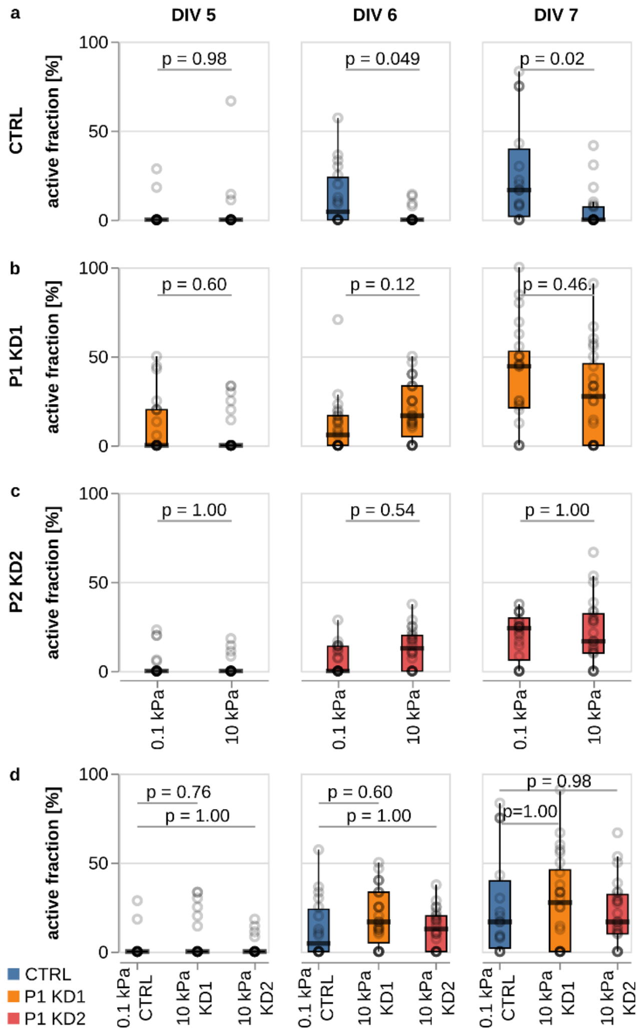

Supplementary Figure 14 | Extended comparison of neuronal activity as a function of substrate stiffness, time in culture, and Piezo1 expression. (a) From DIV 6, the activity of CTRL neurons is significantly higher on soft than on stiff substrates (Kruskal-Wallis test followed by Tukey posthoc test). (b, c) In Piezo1 KD neurons, the activity of the cells is independent of substrate stiffness. (d) The activity of CTRL neurons on soft substrates is similar to the activity of Piezo1 KD cells on stiff substrates, indicating that stiff substrates activate Piezo1, which then slows down electrical maturation of neurons. Data replotted from Fig. 3k. Boxplots show the median (central line), the interquartile range (box), and whiskers represent 1.5 times the interquartile range. Numbers of cells and fields of view are provided in Figure 3 k.

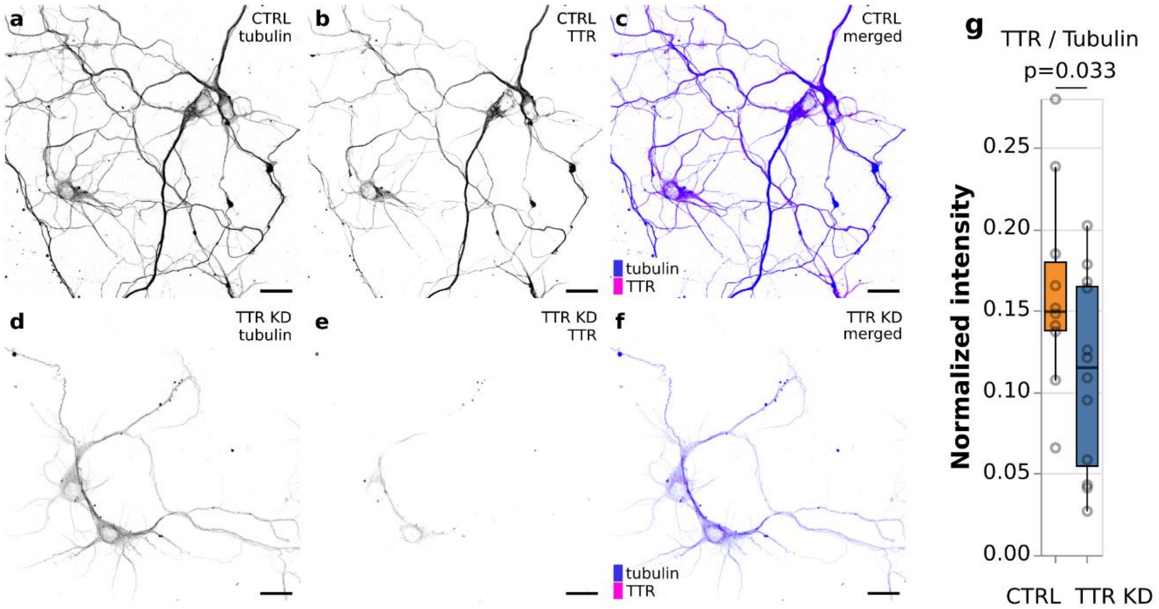

Supplementary Figure 15 | TTR KD in primary neurons. Stainings for individual proteins were intensity-matched between the CTRL neurons (a-c) and TTR KD neurons (d-f). (a) Tubulin staining and (b) TTR staining of CTRL cells (merged in (c)). (d) Tubulin staining and (e) TTR staining of TTR KD cells (merged in (f)). (g) Quantification of fluorescence signals. The total TTR signal per FOV was normalised to the total tubulin signal. We found a significant reduction of TTR in the KD condition (one-tailed t-test). n_{CTRL} = 10 FOV, n_{TTRKD} = 12 FOV. Boxplots show the median (central line), the interquartile range (box), and whiskers represent 1.5 times the interquartile range. Scale bars: 20 µm.

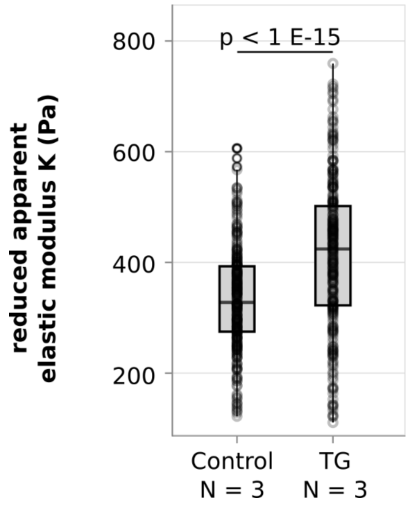

Supplementary Figure 16 | Transglutaminase (TG) treatment increases brain tissue stiffness. Plot shows apparent elastic moduli of *in vivo Xenopus laevis* brain tissue measured by AFM. TG significantly increased tissue stiffness (two-tailed t-test). Boxplots show the median (central line), the interquartile range (box), and whiskers represent 1.5 times the interquartile range.

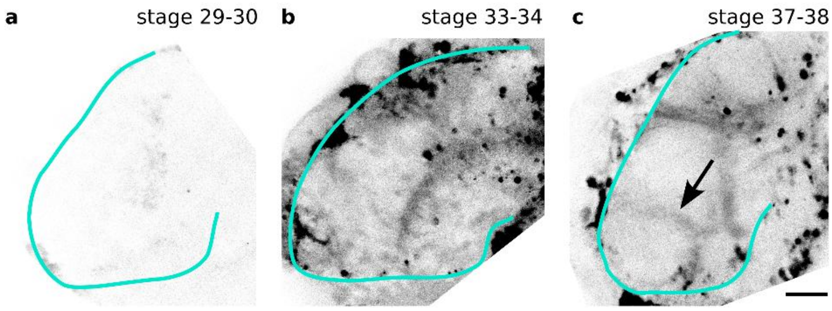

Supplementary Figure 17 | Synapse patterns in developing *Xenopus laevis* brains.

(a-c) Exposed *Xenopus laevis* brains labelled with the synaptic marker FM1-43. (a) At stage 29-30, no synapses are visible yet. (b) At stage 33-34, some first synaptic patterns emerge in the diencephalon. (c) From stage 37-38 onwards, brains of wild type animals showed a characteristic pattern of synapses, including a semicircle surrounding the telencephalon coinciding with the position of the supraoptic tract (arrow). All images are intensity matched. Outlines of brains indicated by green line. Scale bar: 100 µm.

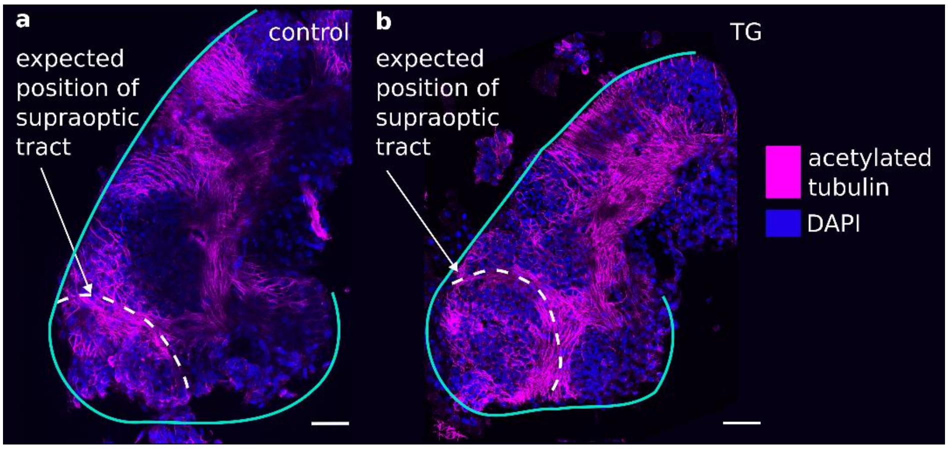

Supplementary Figure 18 | Axon tract formation is unaffected by TG treatment. Acetylated tubulin staining revealed the location of axon tracts. Their characteristic patterns, which form before the start of the treatment[34], are visible in both (a) control and (b) TG-treated brains of stage 37-38 embryos. White dashed curves indicate the areas of the supraoptic tract. Outlines of brains indicated by green curves. Scale bars: 50 µm.
