# Supplementary material for: Environmental stiffness regulates neuronal maturation via Piezo1-mediated transthyretin activity
Source: Nat Commun. 2025 Nov 7;16:9842. doi: 10.1038/s41467-025-64810-3 (PMC12594958; doi:10.1038/s41467-025-64810-3)
Supplement: Supplementary file 2 — Description of Additional Supplementary File [file 41467_2025_64810_MOESM2_ESM.pdf]

Description of Additional Supplementary Files

Supplementary Movie 1 :

Calcium imaging of CTRL neurons on a soft gel at DIV7. Calcium imaging of wildtype control neurons cultured on a soft hydrogel. The majority of cells show peaks. Intensity is colour-coded. Calcium peaks are represented by a change in colour in the corresponding cell. Video is at 4x original speed. Scale bar: 10 μm.

Supplementary Movie 2 :

Calcium imaging of CTRL neurons on a stiff gel at DIV7. Calcium imaging of wildtype control neurons cultured on a stiff hydrogel. Unlike in the other conditions, the cells show no peaks. Intensity is colourcoded. Calcium peaks are represented by a change in colour in the corresponding cell. Video is at 4x original speed. Scale bar: 10 μm

Supplementary Movie 3:

Calcium imaging of P1 KD neurons on a soft gel at DIV7. Calcium imaging of Piezo1 knockdown neurons cultured on a soft hydrogel. The majority of cells show peaks. Intensity is colour-coded. Calcium peaks are represented by a change in colour in the corresponding cell. Video is at 4x original speed. Scale bar: 10 μm

Supplementary Movie 4:

Calcium imaging of P1 KD neurons on a stiff gel at DIV7. Calcium imaging of Piezo1 knockdown neurons cultured on a stiff hydrogel. The majority of cells show peaks. Intensity is colour-coded. Calcium peaks are represented by a change in colour in the corresponding cell. Video is at 4x original speed. Scale bar: 10 μm.

Supplementary Data 1:

Summary of the mapping statistics and number of genes identified for each library.

Supplementary Data 2:

List of all key resources used in the study.
